# The Niagara Meeting

**Published:** 1899-12

**Authors:** 


ARTICLE III.

### THE NIAGARA MEETING.

The meeting of the National Dental Association at Niagara Falls was a magnificent success. The attendance was unusually large and moreover the most prominent men throughout the country were present, and the papers were of a high order.

The Omaha meeting having been such a signal failure it is worth our while in view of the future to inquire into the causes which made the very next meeting so great a success. As contributing causes the popularity of Niagara as a place of meeting, and the fact that the meeting occurred nearer to the large metropolitan cities with their great aggregate of dentists, must of course be reckoned. But the chief credit should be awarded to the president, Dr. H. J. Burkhart. It is true that the section officers did their share, but section officers existed prior to the Omaha meeting; indeed, there has been little change in the personnel; it must be conceded therefore that even the energy of these gentlemen was but a reflex of an impetus originating with the president.

It can be stated that within a week of his election Dr. Burkhart had communicated with his friends demanding their assistance and allegiance in making the Niagara meeting successful. Especially did he point out to the New York men that the National Association had conferred upon them an honor in choosing their state as a meeting place and one of their number as their presiding officer, and he insisted that New York should aim to make the meeting memorable. Within a month the president through his personal solicitation had secured the promise of several important papers, including one from Dr. Jenkins, of Dresden, who came across the ocean to fulfil his pledge in person. Hence with due thanks to all others who worked for the meeting, and there were many, the chief praise must be alloted to the retiring president, who fulfilled all his duties so well and gracefully. The Association is also to be congratulated upon choosing Dr. Holly Smith as its next president, his personal popularity as well as his known perseverance and interest in the welfare of the Association which he helped to organize, being such as to give us promise of another good meeting in 1900.

In addition to the National Dental Association there were also the usual annual meetings of the Examiners and Faculties Association, and it is pleasant indeed to record that these two bodies have at last found a means of settling their differences, as elsewhere reported in this number. This is most opportune, as the time is ripe for great strides forward not only in advancing the standard of admission into the colleges, but for bettering the quality of education afforded. The Faculties being no longer troubled by the antagonism of the Examiners will undoubtedly achieve better things in their college management, and the Examiners having ceased to wrangle with the colleges, may perhaps find time to discover a means of unifying the State laws, or at least of giving us interchange of license, by the plan outlined in our July issue, or by any other which would promise practical results and relieve honest, capable dentists from the nuisance of examination whenever they cross a state line.

At least one of the objects for which we have contended recently in our pages seems to have been achieved; that is the abatement of objectionable college advertising. The Faculty Association adopted a rule forbidding a college to advertise in a manner which would be interdicted by the Code of Ethics in the case of an individual practitioner. This resolution will be found in the report of the Faculties Association meeting in this issue. We have received several communications continuing the discussion of the question of college advertising, but we deem it best to let the subject rest, believing that the Faculties Association passed the new rule in good faith. Should any college see fit to defy the Association and make use of such advertising methods, it would be well for the dentists of the locality to collect a few of the advertisements and forward them to the officers of the Faculties Association that they might make an example of the offender.

There is little doubt that the publication of a long and attractive programme of papers, in advance of the meeting did much to bring about the large attendance, and the crowd once present found compensation for disappointment in the meeting of numerous friends and the making of new acquaintances. Nevertheless it is unfortunate that so many prominent men should have prepared papers for this meeting, only to find that there would be no time when they might be read. Moreover it was deplorable that such papers as were read could not be adequately discussed, as often the discussion of a paper much enhances its value, while not infrequently many things are brought out which are more interesting than the original paper.

The chief trouble seems to have been with the rules of order, or at least with the manner of working under the rules. The scheme of section work is ideal, provided the membership in the sections be sufficiently great so that an essayist would feel contented to read his paper before his section, rather than, as now, to feel aggrieved if his paper be not offered to the full body;

If all papers could be presented to the section first, and if each section were limited to the recommendation of a single paper to be made the feature of one session of the main body, there would be abundant time for the full discussion of all papers, while there would be an incentive to authors to do their utmost, that their work might be honored as the choice of their section.

This method, however ideal, does not seem very feasible in our National Dental Association because of the small membership in some sections.

As an alternate plan, and one which offers more diversity in the National programme, the following is suggested : All papers should be sent to the appropriate sections. They should be read and recommended in the order of their importance or merit, being numbered accordingly. Then when the sections are called, instead of as now, reading all the papers from a single section before proceeding to the next, only the first paper recommended by the first section should be read, after which the first paper from the second section, to be followed by the first paper from the third section, and so on in order, taking one paper from each section until all had been heard, whereupon, there being time, the first section could be called on for its second paper. If it would seem invidious for the sections to discriminate as to the merits of the various papers, another means would be for the sections to report all papers to the Executive Committee, who should arrange the programme following the rule of not calling a second paper from any other section until each of the other sections should have had opportunity to furnish an essayist.

Under the present system, it is possible for one section to contribute a number of dry papers, monotonously descanting on a single subject or slight variation of it, to the exclusion of more interesting papers, which other sections might have to offer. There is a rule, however (if it is an unwritten rule it should be written as soon as possible), that essayists should not require more than half an hour in which to present their subject; nor should it be permitted to offer a written paper of a length within the limit, and then, having the floor, to occupy an hour or more by the addition of extemporaneous remarks; nor should old matter be introduced in whole or in part, except by special permission, given at the time. This rule was broken in all its parts. Some papers offered could not have been read in less than an hour; some were read which occupied more than half an hour; some essayists added extemporaneous remarks, occupying time which might have been accorded to others, or to discussion, and some papers contained old matter, some of which got before the meeting and some of which did not.

Since the International Tooth Crown Company recently stirred up the dentists of the country by announcing through the Associated Press that under a recent legal decision they would now proceed to collect several millions (or was it billions ?) of dollars, we have received numerous letters asking for information. By way of reply we offer the following extract from a circular letter recently received from Dr. Crouse:

"DEAR DOCTOR: You may or may not have heard of the recent decision which relates to the Low bridge patent. To make sure that the members have a full understanding of what has recently transpired, I will review briefly some of the past litigation.

To begin with, the International Tooth Crown Company had originally secured a decision by the highest court in the southern district of New York, declaring some of the various patents on crowns and bridges valid. With these decisions in their favor, as you may remember, eleven years ago they started through the country securing licenses and collecting royalty, the terms imposed being twenty-five dollars ($25) per year license fee and fifteen per cent. on all work done. Those signing a license agreed thereby to the validity of some thirty-eight different patents which the Crown Company had secured on various devices.

At this stage of the proceedings the Protective Association was organized and within six weeks it had stopped the Crown Company from enforcing their patent claims. The Association drove them from one court to another, they withdrawing and paying costs rather than make a test case, until they reached the Federal district in which they had previously obtained their favorable decision. It was here that the Protective Association succeeded in having the former decision of these courts reversed and the Low bridge patent declared invalid; first before the Federal judge in the Circuit Court, and afterward in the United States Court of Appeals. This supposedly to all intents and purposes ended the litigation with the Crown Company.

In the meantime the International Tooth Crown Company had reorganized and secured in their company individuals of large means, and their efforts were being exerted to get a reversal of our decision, on the ground that they had had a favorable decision on their patents at one time, and at the second contest they had been reversed, and so they were entitled to a third hearing. We have known all this and have been pleading, wherever we could get in communication with the members, that this action was being taken by the Crown Company, and we predicted that just the results that have taken place would transpire, namely, that the Crown Company would get a decision in their favor because there was no adequate defense being made. Bear in mind, the defendant was a relative of persons connected with the Crown Company, and not a member of our Association; therefore *the Protective Association has not been represented in any way in the recent suit.* I knew of this action on the part of the Crown Company through some of the former witnesses who had been asked to testify a second time, and especially through personal interviews with the individuals who have recently invested money with the Crown Company, as they wished to know what the position of the Association would be in case of their winning this suit.

They have had their decision by Judge Townsend, which was rendered July 31, 1899, and which practically overrules or reverses the former decision, which declared the patent invalid. With this in their favor the International Tooth Crown Company are already commencing suits and sending communications to the members of the profession, a copy of one of which we append herewith:

WILLARD A. MITCHELL,
COUNSELOR AT LAW,
35 Nassau St.
Telephone 5409 Cortlandt.

NEW YORK, August 5, 1899.

DR. ——————————

DEAR SIR: After several years of litigation, the James E. Low patent (No. 238,940), owned by the International Tooth Crown Company, of this city, has been finally sustained, and the right of said company established to an accounting from all dentists who have been making the Low or so-called "Richmond" bridge.

In corroboration of such information in the matter as has already been furnished by the public press, your attention is invited to the opinion by Judge Townsend in the case of the International Tooth Crown Company vs. Kyle filed in the office of the Clerk of the United States Circuit Court, in this city, on July 31, 1899. Copies of this opinion may be had upon application to this office.

I have been instructed by the said International Tooth Crown Company to prepare and return to it before the end of this month two separate lists of names of the dentists who have not yet settled and who are known to have done work infringing upon said patent; one list to contain the names of those who should be treated with courtesy, and with whom a liberal and amicable adjustment should be made, the other to contain the names of those who fail to respond to my communication or whose replies are unsatisfactory.

I have also been instructed not to institute any legal proceedings involving costs or other expense to defendants until an opportunity has been afforded to respond to this letter by filling in and returning the blank herewith enclosed.

Requesting the favor of an early reply, I am, very truly yours,

WILLARD A. MITCHELL,

Attorney for the International Tooth Crown Company.

This is the blank referred to:

——————————1899.

WILLARD A. MITCHELL, ESQ.,

Attorney for the Int. Tooth Crown Co.,

35 Nassau St., N. Y. City.

DEAR SIR: I elect to settle my indebtedness to the

International Tooth Crown Company for infringement of its patents covering the Low or "Richmond" bridge work upon the basis of the usual license fee, amounting to $25 for each year in which such work was done by me between March 15. 1881, and March 15, 1898, together with a royalty of 15 per cent. on all accounts collected by me for such work between said dates, provided my statement of the amount due is accepted by said company, and provided a discount of 25 per cent, is allowed me by said company for a cash settlement. For the purposes of such an adjustment, and without admitting, except for the purposes of such settlement, that I am indebted in any amount whatever, I state and represent that the total license fee to which said company would be entitled from me during said period, after deducting any amounts which I may have paid for royalties and license fees, does not, to the best of my information and belief, exceed the sum of $ , and that the total amount of royalty doos not exceed the sum of $ , and I hereby offer to adjust my indebtedness to the International Tooth Crown Company by the payment of 75 per cent ef said sums

(Signed)——————

Now, Doctor, the war is on and prompt action is necessary. Heretofore the benefits of the Association have earned to members and non-members alike. The nature of the litigation was such that this could not be very well avoided, that is. the members of the Protective Association have borne all the expenses of the litigation, and while this has not been an enormous burden, since it did not amount to over a dollar a year to each member, nevertheless it was unfair to the members of the Association. Now the litigation takes a different form, and after discussing the whole question very carefully with our attorneys we have decided to protect only the members in good standing who have paid their assessment, and those who do not unite with the Association must take care of themselves. The form that this litigation will assume now will be much more troublesome, involving even more care and responsibility on my part than the former litigation, and I take on this work again very unwillingly and only from a sense of duty, as I can ill afford to neglect my practice, which is my only source of income, to give the time necessary for these additional duties. I do so only with the distinct understanding that the members stand by me, in which case they need have no fear of being compelled to pay royalty. That the Crown Company will collect millions of dollars from the dental profession we have no doubt, but let it be from those who do not come in with us.

The object of this letter is to ask you to send your ten dollars due on the assessment, and we think it hardly necessary to emphasize the need of a prompt response. Make no mistake—when I state that we can protect the members I do so advisably. If the Crown Company make any demands upon you, state that you are a member and refer them to me.

Please let me hear from you by return mail.

Yours very truly,

J. N. CROUSE,

Chairman Dental Protective Association,

2231 Prairie Ave, Chicago."

It has been announced that new members will be received into the Protective Association upon payment of $20, and these new members will be protected.—ED. in *Items of Interest.*

